# A cost-minimisation study of 1,001 NHS Direct users

**DOI:** 10.1186/1472-6963-13-300

**Published:** 2013-08-08

**Authors:** Rod Lambert, Richard Fordham, Shirley Large, Brian Gaffney

**Affiliations:** 1 Health Economics Consulting, Faculty of Medicine and Health Sciences, University of East Anglia, Norwich, Norfolk NR4 7TJ, UK; 2 NHS Direct, Strawberry Fields, Berrywood Business Village, Tollbar Way, Hedge End, Hampshire SO30 2UN, UK; 3 Medical Director, Vhi SwiftCare Clinics, Dublin, Ireland

## Abstract

**Background:**

To determine financial and quality of life impact of patients calling the ‘0845’ NHS Direct (NHS Direct) telephone helpline from the perspective of NHS service providers.

**Methods:**

Cost-minimisation of repeated cohort measures from a National Survey of NHS Direct’s telephone service using telephone survey results. 1,001 people contacting NHS Direct’s 0845 telephone service in 2009 who agreed to a 4-6 week follow-up. A cost comparison between NHS Direct recommendation and patient-stated first alternative had NHS Direct not been available. Analysis also considers impact on quality of life of NHS Direct recommendations using the Visual Analogue Scale of the EQ-5D.

**Results:**

Significant referral pattern differences were observed between NHS Direct recommendation and patient-stated first alternatives (p < 0.001). Per patient cost savings resulted from NHS Direct’s recommendation to attend A&E (£36.54); GP Practice (£19.41); Walk-In Centre (£49.85); Pharmacist (£25.80); Dentist (£2.35) and do nothing/treat at home (£19.77), while it was marginally more costly for 999 calls (£3.33). Overall an average per patient saving of £19.55 was found (a 36% saving compared with patient-stated first alternatives). For 5 million NHS Direct telephone calls per year, this represents an annual cost saving of £97,756,013. Significant quality of life differences were observed at baseline and follow-up between those who believed their problem was ‘urgent’ (p = 0.001) and those who said it was ‘non-urgent’ (p = 0.045). Whilst both groups improved, self-classified ‘urgent’ cases made greater health gains than those who said they were ‘non-urgent’ (urgent by 21.5 points; non-urgent by 16.1 points).

**Conclusions:**

The ‘0845’ service of NHS Direct produced substantial cost savings in terms of referrals to the other parts of the NHS when compared with patients’ own stated first alternative. Health-related quality of life also improved for users of this service demonstrating that these savings can be produced without perceived harm to patients.

## Background

NHS Direct was conceived and implemented in 1999 as a telephone and Internet service through which patients and the public could be ‘signposted’ to the most appropriate health care resources to meet their immediate needs. By 2009 its service was being provided across 36 sites nationally and was taking over 5 million telephone calls alone per year [1]. NHS Direct has since undergone considerable redesign to align its service delivery model to the requirements specified within the wider NHS reforms of the Out of Hours urgent care pathway. It has been replaced by the more comprehensive 111 service. Although the service is restructuring, the economic lessons for similar services can be learned about the efficiency of over-the-phone consultations and referrals from this comprehensive analysis of the UK’s most far-reaching telephone health help-line service to date.

In March 2009, a survey of 3,000 NHS Direct telephone contacts was conducted by a market research company (IFF Research Ltd) on behalf of NHS Direct. In April 2009, 1,001 of these callers who had agreed to be followed up were contacted again.

The main aim of this evaluation was to determine the financial impact, from the perspective of NHS service providers, of patients calling the ‘0845’ NHS Direct telephone helpline. Previous studies of the impact of NHS Direct on demand for other NHS services to date have been equivocal, with examples of both increasing and decreasing cost impacts [2-4]. Correspondingly there has been no clear overall picture of savings or additional costs due to its existence. It is also important to ascertain the impact on the quality of care received arising from NHS Direct referral advice. To demonstrate this we adopted a cost-minimisation approach that ensured any reduction in cost was not at the expense of a poorer outcome. Despite needing more substantiation of such benefits in further trials we have been able to demonstrate that these users of NHS Direct did maintain or improve their overall quality of life.

## Methods

Two standardised telephone surveys of NHS Direct telephone service users were conducted during 2009 by an independent market research company acting on commission from NHS Direct. The first was a survey of 3,000 fifteen-minute telephone interviews conducted in March 2009, of callers to NHS Direct telephone services who had consented to be contacted about their experience of NHS Direct across all regions of England. This initial survey is referred to as the ‘Main stage survey’. In this survey patients explained what referrals had been suggested to them by NHS Direct and 95.3% of patients stated that they followed the NHS Direct recommendation. This was ‘service improvement’ evaluation and did not require full ethics committee approval. Nonetheless we followed a strict ethical code in all aspects of the delivery and conduct of the study.

The range of reported referral options were standardised into seven categories to enable direct comparison between the groups (See Table 1). Patients were also asked about the perceived urgency of their reason for initial contact with NHS Direct and a range of quality of life measures. It should be noted that urgency and quality of life issues could only be undertaken for the actions recommended by NHS Direct, as the first alternative was a hypothetical option within the same population.

**Table 1:** Standard cost tariff


Treat at home/do nothing	£0.00
Pharmacist	£4.00
GP/GP Practice	£19.17
Walk-in Centre	£28.00
Dentist (not leading to admission)	£57.00
A&E attendance	£111.00
999 Ambulance Journey	£344.00

Source: Curtis (2008).

A second stage (‘economic impact survey’) telephone survey was conducted during April 2009 (4-6 weeks after the main stage). This was conducted with 1,001 callers from the main stage survey who had consented to be included in a second survey. This repeated the initial survey questions, including the EQ-5D VAS scale and urgency measures.

Two methods were used to ensure that the survey data used were robust and able to represent the general population. The first method was that data in both surveys were weighted to ensure that the proportion of people within each referral option was representative of the national population of NHS Direct telephone callers. It was felt, however, that these weightings alone would not provide sufficient reliability; therefore a sensitivity analysis was also conducted to examine variance of results around key indicators used. This two-stage approach provides assurances that the reported findings are as robust as possible.

The acknowledged lack of a control group in this survey was addressed by asking callers ‘what service/s, if any, they would have otherwise used had they not called NHS Direct?’ Patients were subsequently followed up at four to six weeks after their initial call to determine the costs and consequences of their referral contact point. A feature of the study was to administer the VAS quality of life question as included in the EuroQol-5D (EQ-5D) by telephone on initial call and again after treatment.

Standard NHS cost tariffs were applied to both the recommendations made by NHS Direct to its callers and to the alternative action that the same callers stated they would have taken if NHS Direct had not been available to them. Although we asked for more than one ‘first alternative’ service (assuming a degree of risk), the additional alternatives were not consistently well answered and could not be sufficiently relied on for further economic analysis. Without a comparison group we had to accept the stated first alternative as being the most likely alternative course of action.

Whilst it would have been desirable to have included NHS Direct’s operating costs, the costs of the ‘0845’ service were under review at the time of the study. Therefore only the gross saving could be estimated. However, it was the scope of the referral savings to the NHS, not the wider value of NHS Direct’s informational role, which was of principal interest here.

The principal analysis was to examine the cost of NHS Direct recommendations compared with patients’ stated first alternative courses of action had NHS Direct not been available. Although they were only within-subject controls by adopting a cost-minimisation framework we were able to show that health-related quality of life was maintained by subjects actually taking up NHS Direct referrals.

Applying a standard tariff to each major referral option, we produced a cost per case from which a cost difference was derived. The cost tariff applied to each of the referral options were taken from the available costs of Health and Social Care 2007/08 [5] at the time of the study. These assumptions are shown in Table 1.

The cost tariff applied was based on rational assumptions of the most likely resource use. For example, the cost of a contact to the GP/GP Practice was estimated using a 7-minute contact time. In addition, the cost of a 999 ambulance journey was based on a fully paramedic trained crew being sent to each contact. This assumption also could not include the outcome from such a call-out as follow-up data were not available. Therefore the assumptions used do not include subsequent hospital costs or the proportion of patients who were treated and resolved by ambulance attendance. Due to these assumptions, sensitivity analyses were performed by increasing the cost of a GP/GP Practice contact from £19.17 to £31 (the cost of an 11-minute consultation) to examine the impact of this assumption on the overall costing analysis. Likewise, the cost of a 999 Ambulance journey was varied between £344 and £263 (the cost without a fully paramedic trained crew). We conflated categories of referral used in the main stage survey and economic impact survey to integrate actual and the stated hypothetical pathways (which were less specifically described by actual users). No discounting of these costs was applied to these estimates as they all occurred within a few weeks after the intervention.

**Figure 1:**
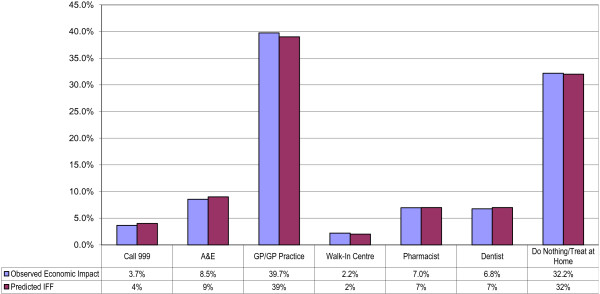
Comparison of predicted and observed weighted proportions of patients with in each treatment option.

The first method used to ensure robustness was that the number of respondents in this survey was adjusted through a weighting procedure to take account of national patterns of NHS Direct use (assessed by previous market surveys outside of this study). The population sampled in this survey was also comparable to that in a similar survey in 2008. We tested that the national weightings applied did in fact lead to a close correlation between predicted and observed proportions of patients within each of the treatment options. We were satisfied that this was indeed the case with less than a 1% variance from the predicted proportion for all options (see Figure 1).

The costs were ‘grossed up’ to be indicative of the total annual cost for 5 million calls per annum and therefore more useful for policy purposes. It should be noted that no such sample size weightings were applied to the quality of life measures, since previous quality of life work had not been undertaken and it was inappropriate to adjust quality of life in this similar way.

### Analysis

Analysis was based on descriptive statistics (frequencies and cross tabulations) from the main dataset, carried out using SPSS version 16. Some limited inferential statistics were used to examine whether change observed was statistically significant. This included Pearson Chi square to examine between group resource use differences; paired t-tests to examine quality of life change and Student’s t-tests to compare Quality of Life change between urgent and non-urgent cases. The outputs from SPSS were transferred into Microsoft Excel 2003 for further analyses during which the standard tariff costs were applied and analysed and from which graphic representations of the data were produced.

## Results

The recommendations provided by NHS Direct were significantly different to the options selected as the first alternative if NHS Direct had not been available (Pearson Chi-Square p < 0.001) (see Table 2 - note that in this table weighted figures have been rounded to whole numbers, and that application of the weighting results in a total of 996 patients, rather than the original unweighted sample of 1001 patients). Around 74% of first alternative options were to A&E or GP Practice compared with around 48% from NHS Direct recommendation. In contrast, around 13% of first alternative options were to ‘do nothing/treat at home’, compared with just over 32% from NHS Direct recommendation.

**Table 2:** Cross tabulation of frequencies (Weighted) of the economic impact survey resource use for NHS Direct recommendation and First alternative

		**First alternative**								
		**Call 999**	**A&E**	**GP/GP Practice**	**Walk-In Centre**	**Pharmacist advice**	**Dentist**	**Do nothing/Treat at home**	**Total**	**%**
**NHS Direct**	**Call 999**	8	14	10	0	0	0	4	36	3.6%
	**A&E**	3	48	24	3	0	0	7	85	8.5%
	**GP/GP Practice**	8	92	205	23	8	0	61	397	39.9%
	**Walk-In Centre**	1	9	6	4	0	0	1	21	2.1%
	**Pharmacist advice**	0	15	35	5	8	0	6	69	6.9%
	**Dentist**	0	18	8	0	0	26	15	67	6.7%
	**Do nothing/Treat at home**	6	105	147	20	9	3	31	321	32.2%
	**Total**	26	301	435	55	25	29	125	**996**	100.0%
	**%**	2.6%	30.2%	43.7%	5.5%	2.5%	2.9%	12.6%	100.0%	

To examine the cost differences between NHS Direct recommendation and the patient’s stated first alternative, it was important to recognise that the costs will be different dependent on the perspective taken within the analysis. To illustrate this difference, the figures for A&E attendance are used.

Table 3 shows that 85.46 patients (the figures are not whole numbers due to the applied weightings and are provided to two decimal places for accuracy of grossing the figures) were recommended by NHS Direct to attend A&E. The standard tariff for A&E attendance was £111 and therefore the cost of the NHS Direct recommendation is 85.46 × £111 = £9485.73. However, if the patient-stated first alternative is examined in the same 85.46 patients, then it can be seen for example that 2.84 would have called 999, 48.26 would have gone to A&E, 23.74 would have attended their GP Practice. By considering all the first alternative options for this group of patients, and costing these using the standard tariff, a total costing is established (for this example, the total cost is £6,876.39). By dividing this total cost by the number of patients, we find that the average cost of those patients’ first alternative options was £80.47. In this example therefore, the cost of the patient-stated first alternative is £30.53 less than attending A&E as recommended by NHS Direct.

**Table 3:** **Cost per patient difference for the 85.46 patients who NHSD Recommended to go to A**&**E**

**Patients stated first alternative was:**	**Frequency**	**Unit Cost**	**Patient stated 1st Alternative Total Cost**		
Call 999	2.84	344	£977.63		
A&E	48.26	111	£5,356.73
GP/GP Practice	23.74	19.17	£455.12
Walk-In Centre	3.04	28	£85.02
Pharmacist	0.47	4	£1.89	**Cost-per patient difference**	
Dentist	£0.00	£57.00	£0.00	NHS Direct Recommendation to attend A&E - Cost per patient	£111.00
Do nothing/Treat at home	£7.10	£0.00	£0.00	Patient stated 1st Alternatives - Cost per patient	£80.47
**Total Patients Recommended by NHS Direct to attend A&E**	**85.46**			**Cost per patient difference**	**£30.53**
**Cost per A&E visit**	£111.00				
**NHSD recommendation Total Cost**	**£9,485.73**	**Total cost of Patient stated alternative options to NHS Direct recommendation**	**£6,876.39**		

However, if the analysis is undertaken from the perspective of the patient-stated first alternative of attending A&E (and comparing the costs with those of the NHS Direct recommendations made to this group of patients), then a different result is observed. Table 4 shows that, from this perspective, the total cost of the 300.7 patients who stated they would have attended A&E is 300.7 × £111 = £33,377.45. This compares with a total of £13,208.24 for the range of options recommended by NHS Direct to those patients. The NHS Direct recommendation is therefore £67.07 per patient less costly than the patient-stated first alternative if NHS Direct had not been available.

**Figure 2:**
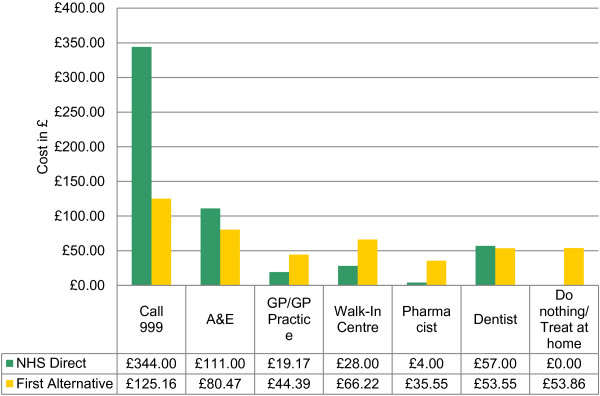
Weighted cost per patient of NHS Direct recomm endation compared with First Alternativ.

By reviewing each of the other referral options similarly, a comprehensive view of the costs from each perspective can be obtained. Figure 2 shows a summary of these costs from the perspective of NHS Direct’s recommendation compared with patients’ stated first alternative. From this perspective, NHS Direct is more costly than the first alternative for 999 calls, A&E and dentist appointments and less costly for the other options.

**Table 4:** **Cost per patient difference for the 300.70 patients who stated they would have gone to A**&**E if NHS Direct had not been available**

**NHS Direct Recommendation:**	**Frequency**	**Unit Cost**	**NHS Direct Recommendation: Total Cost**		
Call 999	13.85	344	£4,765.37		
A&E	48.26	111	£5,356.73		
GP/GP Practice	92.29	19.17	£1,769.21		
Walk-In Centre	9.05	28	£253.27		
Pharmacist	14.58	4	£58.31	**Cost-per patient difference**	
Dentist	17.64	£57.00	£1,005.35	NHS Direct Recommendation to attend A&E - Cost per patient	£43.93
Do nothing/Treat at home	105.04	£0.00	£0.00	Patient stated 1st Alternatives - Cost per patient	£111.00
**Total Patients who state they would have attended A&E if NHS had not been available**	**300.70**			**Cost per patient difference**	**£67.07**
**Cost per A&E visit**	£111.00				
**NHSD recommendation TotalCost**	**£33,377.45**	**Total cost of Patient stated alternative options to NHS Direct recommendation**	**£13,208.24**		

**Figure 3:**
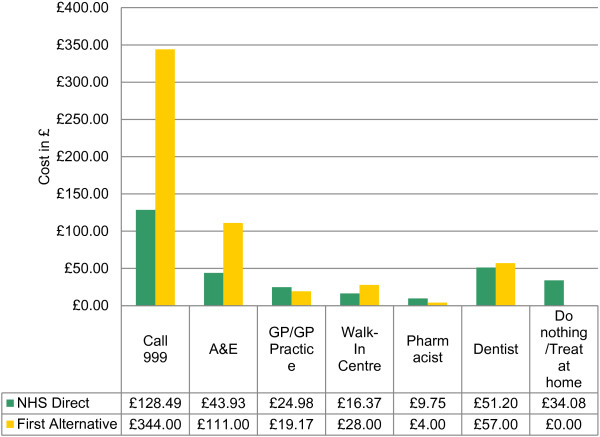
Weighted cost per patient of First alternative compared with NHS Direct reommendation.

Alternatively, Figure 3 shows a summary of costs from the perspective of the patient’s stated first alternative compared with NHS Direct recommendation. From this perspective, with the exception of Walk-in Centre, the trends from Figure 2 are reversed.

**Figure 4:**
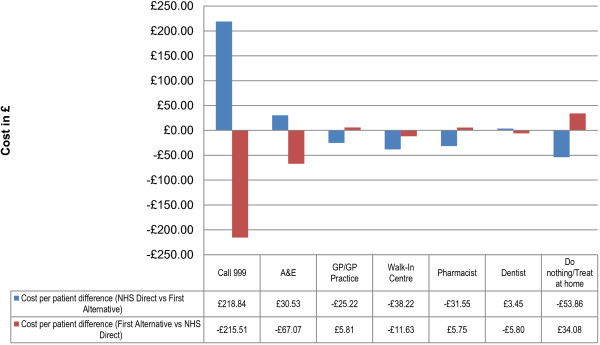
Cost per patient difference.

Figure 4 shows the cost differences per patient from each perspective for each referral option. For example, Figure 2 shows that the cost difference per patient for attending A&E is £30.53 more costly when taking NHS Direct recommendation, and Figure 3 shows this to be £67.07 less costly when taking NHS Direct recommendation.

**Figure 5:**
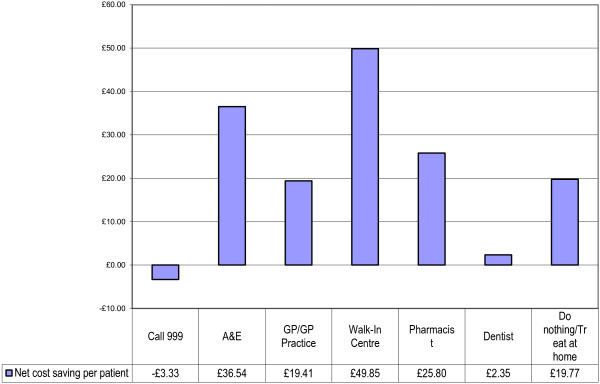
Net cost saving per patient.

By representing the two perspectives together, it is possible to consider the actual cost differences for each referral option (See Figure 5). By considering the costs from the perspective of the overall cost differential from the previous analyses, an overall cost increase is observed for patients following NHS Direct recommendation for calling 999 (£3.33 per patient) and up to £49.85 cost saving per patient with referral to a Walk-in Centre. By taking this broader view of the data, the total costs/savings of each referral option can be identified.

**Figure 6:**
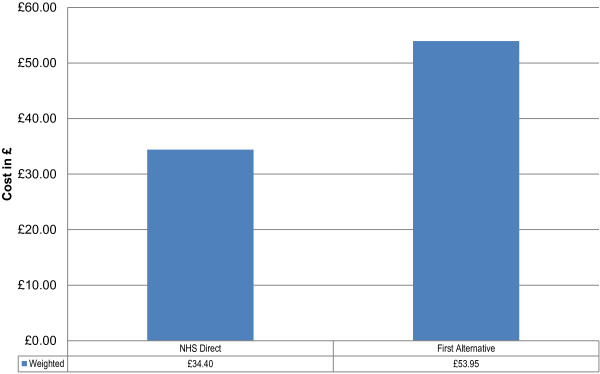
Total weighted cost per patient.

Figure 6 shows that, across all referral options, NHS Direct costs £34.40 per patient, compared with £53.95 per patient for the patient-stated first alternative. The difference between following NHS Direct recommendation and a patient-stated first alternative is therefore £19.55 per patient, representing a 36% cost saving per patient. Assuming that these per patient cost savings from a survey of 1,001 current users are representative across the 5 million telephone contacts received per year by NHS Direct, the overall cost saving is £97,756,013.

As stated earlier, since these savings are highly dependent on unit cost assumptions, the second method to ensure robustness was to conduct a sensitivity analysis. The multi-way sensitivity analysis (see Table 5) varied the costs of 999 calls between £344 (the cost of a fully paramedic trained crew) and £263 (the cost of a more basically trained crew). It also varied the cost of GP Practice attendance between £19.17 and £31.00 to account for different levels of GP time.

**Table 5:** Sensitivity analysis

						**Variance from the average**			
	**999 = £344 + GP = £19.17**	**999 = £263 + GP = £19.17**	**999 = £344 + GP = £31**	**999 = £263 + GP = £31**	**Average annual cost savings**	**999 = £344 + GP = £19.17**	**999 = £263 + GP = £19.17**	**999 = £344 + GP = £31**	**999 = £263 + GP = £31**
Mainstage Unweighted	£144,391,905.35	£140,861,394.77	£154,360,730.59	£150,830,220.01	£147,611,062.68	97.8%	95.4%	104.6%	102.2%
Mainstage Weighted	£97,509,876.63	£101,684,902.29	£98, 708, 201.64	£102,883,227.31	£100,196,551.97	97.3%	101.5%	98.5%	102.7%
Economic Impact Unweighted	£110,594,055.94	£108,166,483.52	£118,216,783.22	£115,789,210.79	£113,191,633.37	97.7%	95.6%	104.4%	102.3%
Economic Impact Weighted	£97,756,012.97	£102,066,686.63	£100,031,550.35	£104,342,224.00	£101,049,118.49	96.7%	101.0%	99.0%	103.3%
Unweighted Average	£127,492,980.65	£124,513,939.14	£136,288,756.91	£133,309,715.40	£130,401,348.02				
Weighted Average	£97,632,944.80	£101,875,794.46	£99,369,875.99	£103,612,725.66	£100,622,835.23				
Mainstage Unweighted % from average	113.3%	113.1%	113.3%	113.1%					
Economic Unweighted % from average	86.7%	86.9%	86.7%	86.9%					
Unweighted Variance from the average	26.5%	26.3%	26.5%	26.3%					
Mainstage weighted % from the average	99.9%	99.8%	99.3%	99.3%					
Economic weighted % from the average	100.1%	100.2%	100.7%	100.7%					
Variance	0.3%	0.4%	1.3%	1.4%					

The sensitivity analysis also considered the effect of the applied weightings and included analysis of unweighted values and of both main stage and economic impact datasets. This sensitivity analysis showed that the weighted data produced less variance than the unweighted datasets and that the costing assumptions used in the main analysis also produced the lowest variance compared with the additional varied assumptions. Therefore we consider the cost saving estimates presented to provide the most robust analysis.

### Quality of Life

**Figure 7:**
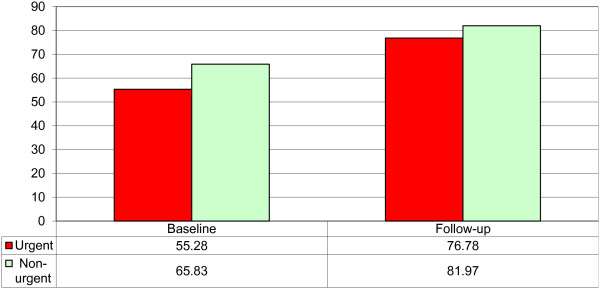
Self-related quality at baseline and follow-up by level of urgency.

Having estimated the costs, patient reported quality of life outcomes were examined using the EQ-5D VAS rating scale (0-100), guided by a telephone survey team. These were completed by 471/1001 (47%) participants at baseline, and 469/1001 (47%) at follow-up. Results showed that mean baseline score was 57.37 points, and improved to a mean of 77.5 at follow-up, an overall improvement of 20.1 points. A paired t-test showed a highly significant difference (p < 0.001; 95% CI = 18.42 to 23.95) between baseline and follow-up (See Figure 7).

When viewing overall health status from the perspective of level of urgency, people who reported that they considered their problems as urgent or non-urgent gained between 16.1 (non-urgent) and 21.5 points (urgent) between baseline and follow-up. This represented a significant ‘between group’ difference at both baseline (p = 0.001; 95% CI = -15.63 to -2.96) and follow-up (p = 0.046; 95% CI = -10.28 to -0.09), although the between group differences were smaller at follow-up.

**Figure 8:**
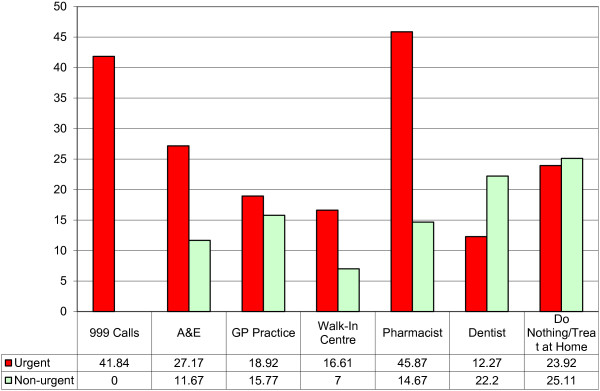
Change in mean self rated health status between baseline and follow-up by urgency.

When considering the change in mean self-rated health status between baseline and follow-up for each referral option and by perceived urgency (see Figure 8), the greatest improvement is achieved from urgent referrals to Pharmacist (45.87 points), 999 calls (41.84 points), and A&E (27.17 points). Figure 8 also shows the difference between urgent and non-urgent cases and in two of the seven referral options urgent cases improve more than non-urgent cases. In only two of the referral options (Dentist and Do nothing/treat at home) is this trend reversed, with non-urgent cases improving more than urgent ones.

It was not possible to calculate an incremental cost-effectiveness ratio (ICER) in this study (as no control group actually existed to compare incremental benefits). However, as both costs reduced and outcomes improved significantly the NHS Direct alternative can be considered a ‘dominant’ option.

## Discussion

### Statement of principal findings

From an analysis of 1,001 users of the service, the cost of NHS Direct recommendations across all referral options was £34,430 compared with a total cost of the patient-stated first alternative of £54,000. This is an overall cost saving of £19,571 for this sample population. The cost per case of the sample population following NHS Direct recommendation was £34.40 compared with £53.95 for the patient-stated first alternative, a cost saving of £19.55 (36%) per patient. When this per patient cost saving is applied to the 5 million calls received by NHS Direct annually, the total cost savings produced would be at least £97,756,013. The sensitivity and variance analyses conducted demonstrated this was a conservative, but robust estimate.

However, quality of life is clearly an important consideration when discussing effects of interventions in established clinical pathways (and ultimate treatment patterns that arise from these) and this must be weighed carefully alongside any savings in resource use. The current analyses have shown that overall the referral options recommended by NHS Direct resulted in statistically significant positive changes in quality of life between baseline and follow-up. Clearly, what is still uncertain is whether these improvements would have occurred anyway, either as a result of following conventional referral pathways or through a process of natural recovery, or both.

When looking at cases perceived by the patient to be urgent or non-urgent (please note that 10 patients responded with ‘Do not know’), there are more patients contacting NHS Direct who consider their situation as urgent (800 patients) than non-urgent (191 patients). In terms of self-rated health status, recorded using a 0-100 scale, statistically significant between group differences were observed at baseline (Urgent = 55.28; Non-urgent = 65.83; p = 0.001), showing that urgent cases initially had lower health status than non-urgent cases. At follow-up, the differences between urgent and non-urgent cases had reduced, but remained statistically significant (Urgent = 76.78; Non-urgent = 81.97; p = 0.046), although the difference was smaller at follow-up. Overall, this shows that urgent cases improved by a mean of 21.5 points, compared with 16.1 points for non-urgent cases.

When looking at the impact of the referral options most often recommended by NHS Direct, patients referred to a pharmacist, 999 call or A&E gained the most benefit in terms of self-reported health status between baseline and follow-up. However, non-urgent cases who are recommended to go to the dentist or to ‘do nothing/treat at home’ improved more than urgent cases.

The study has shown both a significant cost reduction and an improvement in quality of life through the telephone advice given by NHS Direct making this service a cost-effective one. However, the findings still require further investigation through a prospective study with a true (rather than hypothetical) control group.

### Strengths and weaknesses of the study

Most importantly, we did not have any formal control group against which to view whether the level of change results from the recommendations of NHS Direct. We acknowledge that the present analyses rest on the hypothetical comparison of what NHS Direct users said they would have done if NHS Direct had not be available.

The cost assumptions used in these analyses have, of necessity, omitted consideration of the outcomes resulting from either accepting NHS Direct recommendation, or the patient-stated first alternative. This may under-estimate costs as it does not therefore include, for example, call-outs by a GP or Ambulance crew who resolve problems without transfer to hospital, compared with those that require further hospital-based treatments. Likewise, the relative difference in costs of patients attending A&E are acknowledged to be different depending on whether they are treated and sent home or assessed and admitted. The sensitivity analyses conducted focused on the elements where reasonable alternative cost-assumptions were possible. However, it is also felt that cost assumptions made have erred on the side of caution and remained as robust as possible within the limitations of available data.

The robustness of analyses has been assured through a rigorous approach using both population weightings and sensitivity analysis. During the sensitivity analysis, both weighted and un-weighted findings were considered, along with variation in relevant costing assumptions. The final reported findings are based on the most robust combination.

Also from the initial survey a population of slightly less than 50% completed the quality of life elements. This led to difficulties in terms of the size of some sub-group analyses which were too small to undertake adequate analyses.

Consideration of the quality of life and urgency issues could only be undertaken from the perspective of NHS Direct recommendation, as any attempt to consider these issues from the perspective of the patient-stated first alternative would have remained hypothetical and would have been from the same sample population. Therefore it was only possible to review this data to examine whether improvement occurred between the main stage and economic impact surveys.

The lack of any case-mix adjustment, apart from the reported urgent/non-urgent dichotomy, is a potential weakness of this study. However, this was controlled by the within-subject design. Also, the sample size did not allow case-mix cost variation to be identified whilst the survey method (of telephone delivery) made it difficult to determine an actual diagnosis. Hence the results should be treated cautiously if intending to apply them to any specific group of telephone users. Future studies should carefully consider case-mix issue.

The analysis did not include any indication of appropriateness. This would have required a much bigger study. For example, If NHS Direct recommended going to A&E, and the first alternative was to do nothing, we did not know how serious the problem really was and what implications (such as a worsening condition) could have arisen from the ‘do nothing’ option, or from attending A&E (such as being admitted, etc.).

### Strengths and weaknesses in relation to other studies (discussing important differences in results)

Early in its development, there was recognition that NHS Direct required rigorous evaluation [6]. Such evaluation was recommended to address the five key issues below:

•Ensure NHS Direct is safe and effective

•To develop National Standards

•To become an integral part of the NHS

•To ensure equity of access for all

•To provide a range of systems for access [6]

The current study had a key bearing on the third of these and has concluded in a preliminary way that integration into the NHS has taken place at least as far as being able to refine the initial point of NHS contact at less cost and without damage to immediate health outcomes.

One of the first formal evaluations of NHS Direct was an observational study, identifying that the service had received about 68,500 calls in its first year [4]. This initial study concluded that NHS Direct had restrained increasing demand on General Practitioner out-of-hours services. Another study a year later identified that levels of urgency was often not recorded, and that a quarter of calls related to 0-5 year olds [7]. At about the same time, unintended consequences were being noted, in that while advice calls to A&E were observed to have significantly reduced, calls to the main hospital increased [8].

One of the first cluster randomised trials of NHS Direct [9] identified that NHS Direct triage was more costly than practice based triage. However, no formal economic evaluations of NHS Direct have been reported in peer-reviewed journals.

Many of the studies of NHS Direct were conducted in its earlier period of development and this current study provides evidence of its use and cost-effectiveness in a mature phase after ten years of existence. The current study shows that around 40% of patients were recommended by NHS Direct to go to their GP Practice and around the same proportion would have elected to go to the GP Practice had NHS Direct not been available. However, there were almost three times as many people recommended by NHS Direct to ‘do nothing/treat at home’, than would have taken this option without NHS Direct. This indicates that the demand management value of NHS Direct on general practice at least remains high even after ten years.

The fact that we have undertaken evaluation by level of urgency shows that the critique by Payne et al (2005) [7] has been addressed. Future studies will need to stratify patient user groups in similar and perhaps more refined ways if the economic impact is to be more carefully interpreted. Another factor is that Payne et al (2005) [7] suggested that 25% of calls to NHS Direct were for children 0-5 years. Our current analysis does not provide a comparable age grouping, but shows that nearly half (44.8%) of calls were on behalf of children under 16 years of age.

Despite earlier indications [9] that NHS Direct was more costly than practice based triage, the current analysis shows that before its own costs are excluded NHS Direct recommendation is £19.55 less costly to the NHS than the patient-stated first alternatives.

### Meaning of the study; possible explanations and implications for clinicians and policy makers

The current study used National Survey data to examine the financial and quality of life implications of NHS Direct telephone recommendation when compared with patients’ own statements about what alternative actions they would have taken had NHS Direct not been available. The outcomes from this analysis show NHS Direct is making substantial patient-level cost savings compared with their likely service use if NHS Direct was not in place. It also shows an observed improvement in quality of life between the main stage and follow-up surveys. However it could be argued that this quality of life profile change would be observed anyway given the natural recovery process from some acute health episode. Therefore, quality of life changes require further examination, although the initial conclusion is that NHS Direct overall improves both quality of life and cost over stated alternatives.

#### Unanswered questions and future research

Major questions unanswered by the current study are related to whether the cost savings and quality of life improvements can be observed if there was a genuine, rather than a hypothetical, control group? This requires a larger, well designed and well funded research study also consisting of subjects who may not have decided to use NHS Direct.

Further consideration should be given to undertaking more detailed research with the following features:

A prospective study design for more accurate cost estimation.

A real, rather than hypothetical, matched comparison group of patients who did not use NHS Direct (but could have done).

A well designed cohort study, comparing the cost-effectiveness of NHS Direct recommendation against an age-, gender and condition-matched control by each major referral recommendations by NHS Direct.

## Conclusions

Studies of NHS Direct’s impact on demand for other NHS services to date have been equivocal, with examples of both increasing and decreasing demand effects as a result of early intervention in the patient referral pathways [2-4]. Therefore there has been a lack of clarity as to the economic implications of the service and no clear overall picture of savings or additional costs due to its existence. In recent years there appears to have been an increasing trend in the use of NHS Direct services, including the ‘0845’ number, which now stands at 5 million calls per annum.

Although these are early economic results, they show some robust impacts on pattern of NHS contact compared to what patients said they would otherwise have done without NHS Direct. This study of 1,001 individuals showed a significant reduction in potential use of A&E and GP visits by about 25% and a 10% increase in patients who decided to do nothing/treat at home following NHS Direct advice. These changes caused an overall reduction in the expenditure on initial NHS contacts of the equivalent of about £20 per call, or a 36% saving in referral cost overall. Some savings were higher, such as those that would have been incurred at Walk-in Centres (£50 per patient) or those visiting a Pharmacist (£26 per patient). Obviously, if the average magnitude of saving can be extrapolated to every NHS Direct caller then the potential for savings is large and will grow with further usage.

We found evidence that the health status of users of NHS Direct (as measured by the VAS scale of the EQ-5D), showed significant improvement. This should offer much greater reassurance to clinicians and policy makers that patients can be dealt with initially equally as well but at significantly less cost by NHS Direct compared to conventional pathways.

### Data sharing statement

Study data has not been made publicly available at this stage due to the competitive nature of the market at present. Specific requests for access to study data can be made to the corresponding author.

### Ethics approval

The Chair of the University of East Anglia/NHS Ethics committee deemed this was ‘service evaluation’ and did not require full ethics committee approval. Nonetheless we followed a strict ethical code in all aspects of the delivery and conduct of the study.

## Competing interests

This study was conducted as a private consultancy agreement between Prof. Ric Fordham and Dr Rod Lambert (the consultancy team) and NHS Direct. All analyses were conducted independently and without influence from NHS Direct. The final report was presented to NHS Direct in 2009 with the delay between the report presentation and publication being caused by service commissioning issues, along with delays in development of the manuscript.

## Authors’ contribution

RF and RL were the main contributors to data analysis and writing of the article. SL and BG contributed to the drafting of the article and ensured that specific detail relating to service elements were accurate. All authors contributed to the final manuscript and approved the final version of the article.

## Pre-publication history

The pre-publication history for this paper can be accessed here:

http://www.biomedcentral.com/1472-6963/13/300/prepub
